# A Single Mutation N166D in Hemagglutinin Affects Antigenicity and Pathogenesis of H9N2 Avian Influenza Virus

**DOI:** 10.3390/v11080709

**Published:** 2019-08-02

**Authors:** Fang Jin, Xiaomei Dong, Zhimin Wan, Dan Ren, Min Liu, Tuoyu Geng, Jianjun Zhang, Wei Gao, Hongxia Shao, Aijian Qin, Jianqiang Ye

**Affiliations:** 1 Key Laboratory of Jiangsu Preventive Veterinary Medicine, Key Laboratory for Avian Preventive Medicine, Ministry of Education, College of Veterinary Medicine, Yangzhou University, Yangzhou 225009, China; 2 Jiangsu Co-innovation Centre for Prevention and Control of Important Animal Infectious Diseases and Zoonosis, Yangzhou 225009, China; 3 Joint International Research Laboratory of Agriculture and Agri-Product Safety, the Ministry of Education of China, Yangzhou University, Yangzhou 225009, China; 4 Institutes of Agricultural Science and Technology Development, Yangzhou University, Yangzhou 225009, China; 5 College of Animal Science and Technology, Yangzhou University, Yangzhou 225009, China; 6 Sinopharm Yangzhou VAC Biological Engineering Co. Ltd., Yangzhou 225127, China

**Keywords:** H9N2, escape mutant, HA, N166D, antigenicity, pathogenesis

## Abstract

Some immune escape mutants of H9N2 virus and the corresponding mutations in hemagglutinin (HA) have been documented, but little is known about the impact of a single mutation on the antigenicity and pathogenesis of H9N2. In this study, seven critical sites in HA associated with the antigenicity were identified and the effects of a HA mutation (N166D) derived from a H9N2 escape mutant (m3F2) were investigated. Although N166D did not significantly affect viral replication in Madin–Darby canine kidney (MDCK) cells and viral shedding in the larynx and cloaca of chicken, N166D attenuated the pathogenesis of the virus in mice. Compared to the rescued RgPR8-H9_166D, RgPR8-H9_166N caused greater body weight loss and higher viral titers in the lungs of the infected mice. Moreover, hemagglutination inhibition (HI) assay for the sera from the chickens infected with wild type H9N2 and mutant m3F2 showed that N166D mutation could result in weak antibody response in chickens. Considering the field strains of H9N2 with N166D mutation are frequently isolated in the countries with H9N2 vaccination, the findings that the single mutation in HA, N166D, affected both the antigenicity and pathogenesis of H9N2 highlight the significance of surveillance on such mutation that may contribute to the failure of H9N2 vaccination in the field.

## 1. Introduction

H9N2 avian influenza virus (AIV) is endemic in Asia, the Middle East, and North Africa, which causes a significant economic loss to the poultry industry due to mild to high morbidity and mortality [1,2,3]. Moreover, H9N2 can offer internal genes to non-H9N2 AIVs, including H5N1, H5N6, H7N9, and H10N8, which are known to cause high mortality in human [4,5,6,7]. Owing to the threat posed by H9N2 to poultry and human health, H9N2 vaccination strategy has been employed in several countries. However, H9N2 still can be frequently isolated from the vaccinated chicken flocks, which is possibly due to antigenic drift [8,9,10,11]. Antigenic drift is frequently caused by accumulative mutations in the hemagglutinin (HA) gene [12]. Therefore, to identify the antigenic sites in HA and evaluate their impacts on the virus is critical for H9N2 surveillance and vaccine design. Some antigenic sites in H9 have been identified [13,14,15,16,17,18], the discovery of new antigenic amino acids may facilitate the understanding of their roles in the antigenicity and pathogenesis of H9N2 influenza virus. In this study, we identified several novel B cell epitopes in HA of H9N2 and found that a single mutation N166D in HA could alter the antigenicity and pathogenesis of H9N2.

The identification of novel antigenic sites broadens our knowledge for fully mapping the B cell epitopes in HA of H9N2, and that the single N166D mutation derived from an escape mutant could efficiently result in low antibody response in chickens highlights that such a mutation may contribute to the failure of H9N2 vaccination in the field and the efficient surveillance on such mutation in field strain is critical for better H9N2 vaccine development.

## 2. Materials and Methods

### 2.1. Virus and Cells

The H9N2 isolate A/chicken/Jiangsu/XZ299/2016/H9N2 (XZ299) used in this study was propagated in 10-day-old embryonated chicken eggs. Madin–Darby canine kidney (MDCK) cells were maintained in Dulbecco’s modified Eagle’s medium (DMEM), containing 5% fetal calf serum (FCS). Human embryonic kidney 293T cells were maintained in Dulbecco’s modified Eagle’s medium (DMEM) containing 10% fetal calf serum (FCS). These cells were incubated at 37 °C under 5% CO_2_.

### 2.2. Monoclonal Antibodies (MAbs)

H9-specific monoclonal antibodies (MAbs) were generated through the fusion of myeloma Sp2/0 cells with splenocytes from Balb/c mouse immunized with XZ299 virus as previously described [19]. In brief, the mice were immunized with XZ299 by three times of intraperitoneal inoculations at 2-week intervals including a final boost on day 3 before fusion. Hybridomas were screened by indirect immunofluorescence assay (IFA) using Madin–Darby Canine Kidney (MDCK) cells infected with XZ299 as antigen, followed by identification with a hemagglutination inhibition (HI) assay. The ascitic fluids of the positive hybridomas were generated in mice as previously described [19].

### 2.3. HI (Hemagglutination Inhibition) Assay

HI assay was performed using 8 hemagglutination units of H9N2 and 0.5% chicken erythrocytes by standard methods as previously described [20].

### 2.4. Selection of Escape Mutant

Selection of escape mutant was described previously [21]. In brief, the ascitic fluid of each MAb was reacted with XZ299 for 30 min at 37 °C and the mixture was then inoculated into 10-day-old embryonated chicken eggs. The allantois fluid from the inoculated eggs was collected and tested by HA and HI assay with MAbs. Those viruses that were not inhibited by corresponding MAbs were selected and the HA genes of these viruses were sequenced as previously described [22].

### 2.5. Recombinant Viruses Rescused by Reverse Genetics

Viral RNAs of XZ299 and H9N2 mutant were extracted and cDNA was synthesized as previously described [22]. The HA (GenBank accession No: MN227199) and NA(Neuraminidase) (GenBank accession No: MN227201) genes from XZ299 and the HA gene from H9N2 mutant were amplified and cloned into the linear influenza vector pDP2002 by the ExnaseTM II, provided by ClonExpressTM II kit (Vazyme Biotech Co., Ltd., Nanjing, China), as previously described [23]. Two recombinant viruses designated as rgPR8-H9 166N and rgPR8-H9 166D were rescued by transfection in the cocultured 293T and MDCK cells as previously described [23]. Briefly, 1 μg of the HA plasmid derived from XZ299 and H9N2 mutant respectively, and 1 μg of NA, NP, PB1, PB2, PA, MP, and NS plasmid derived from PR8 (A/Puerto Rico/8/34 (H1N1)) each were first mixed in 250 μL of Opti-MEM medium and then mixed with 16 μL of TransIT^®^-LT1 Transfection Reagent (Mirus Bio LLC, Madison, WI, USA). The mixture was incubated at room temperature for 45 min, and then 1 mL of Opti-MEM medium was added. The mixture was then inoculated onto the co-cultured 293T and MDCK cells. After 12 h post-transfection, the medium was changed with 2 mL of fresh Opti-MEM medium with 1μg/mL TPCK-Trypsin. At day 4 post-transfection, the rescued viruses in the supernatant of the transfected cells were collected and titrated in MDCK cells.

### 2.6. Viral Growth Kinetics

MDCK cells were infected with rgPR8-H9 166N, rgPR8-H9 166D, XZ299, and m3F2 at 0.001 MOI (Multiple of infection), all at 37 °C. The tissue culture supernatants from the infected cells were collected at 12, 24, 36, 48, 60, and 72 h post-infection (hpi), and the viruses were titrated by TCID_50_ in MDCK cells as previously described [22].

### 2.7. Mice Study

Twelve six-week-old Balb/c mice per group were infected with rgPR8-H9 166N, rgPR8-H9 166D, XZ299, and m3F2 at 10^5^ TCID_50_ by intranasal inoculation respectively. At 3 and 6 days post-infection (dpi), three mice from each group were euthanized, and lungs were collected and the virus load in the lungs were titrated by TCID_50_ in MDCK cells. Six infected mice per group were monitored daily for body weight loss and any clinical signs. The mice with the body weight loss more than 25% will be anesthetized and euthanized.

### 2.8. Chicken Study

Two-week-old chickens were infected with XZ299 and m3F2 at 10^5^ TCID_50_ by intranasal inoculation. At 2, 4, 6, 8, and 10 dpi, the swab samples of the larynx and cloaca from six chickens each group were collected and titrated for virus shedding in MDCK cells. At 7 and 14 dpi, six chickens in each group were bled to determine HI titers against XZ299 and H9N2 mutant, respectively.

### 2.9. Statistical Analysis

Experimental groups were statistically compared using Prism 5.0 software package (GraphPad Software, La Jolla, CA, USA) to perform an analysis of variance (ANOVA). *p* < 0.05 was considered as a statistically significant difference.

### 2.10. Ethics Statement

All animal experiments were performed in accordance with institutional animal care guidelines, and the protocol, #06R015, was approved by the Animal Care Committee at Yangzhou University in China. The ethical permission code (No. SYXY-18, licensed on March 05, 2019) was provided by Animal Ethics Committee of Yangzhou University.

## 3. Results

### 3.1. Four Immune Escape Mutants were Generated by Three MAbs against H9N2

Following selection of MAbs, three MAbs (2G10, 3F2, and 5C7) were generated, and ascetic fluids of the MAbs from mice were subsequently produced and harvested. All three MAbs inhibited XZ299 at high HI titers (Table 1), suggesting that the MAbs recognize the globular head regions of H9. To identify the epitopes recognized by these MAbs, selection for escape mutants of XZ299 was performed with the MAbs in 10-day-old embryonated chicken eggs as previously described [21]. Four H9N2 mutants (m2G10-1, m2G10-2, m3F2, and m5C7) were found (two escape mutants from selection with 2G10 MAb). As shown in Table 1, the mutants were poorly inhibited in HI assay with the selected MAbs, or even no inhibition at all was detected.

### 3.2. Seven Critical Sites Associated with Antigenecity were Identified in HA of H9N2

**Figure 1:**
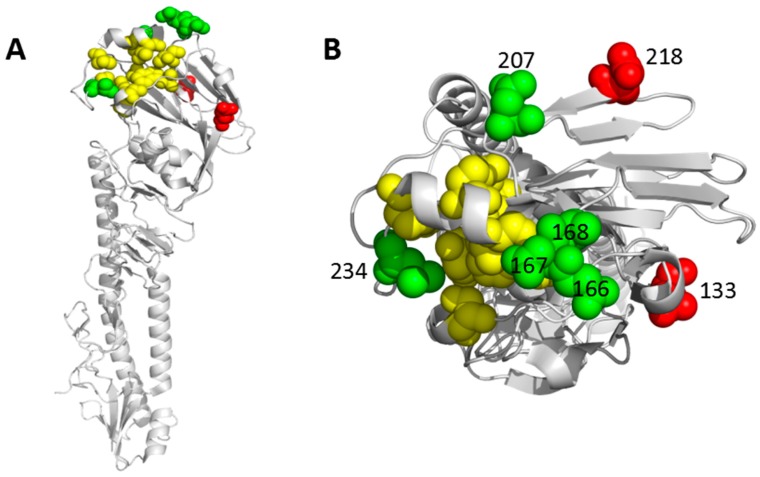
Locations of seven critical amino acid positions in HA of H9. The location of the identified seven mutations Q133L, N166D, N167K, A168D, D207N, N218D, and L234M derived from H9N2 escape mutants was analyzed by Pymol software (Delano Scientific) and shown with the side view (**A**) and top view (**B**) of the locations of these positions on an H9 monomer (PDB ID 1JSD). The two novel positions identified in this study are highlighted in red, while the remaining five are in green. Ten conserved and variable residues (Y109, S148, W161, T163, N193, P194, V198, L202, L234, and G236) (H9 numbering) involving in receptor binding [24] are colored with yellow.

To identify novel antigenic sites in HA of H9N2, the HAs of the H9N2 mutants m2G10-1, m2G10-2, m3F2, and m5C7 were sequenced. As shown in Table 1, three mutants had multiple mutations each, i.e., Q133L/D207N/N218D/L234M in m2G10-1 mutant, Q133L/A168L/D207N/N218D in m2G10-2 mutant, and Q133L/N167K/D207N/N218D in m5C7 mutant. In contrast, the m3F2 mutant identified with 3F2 MAb only had a single mutation, namely, N166D. As shown in Figure 1, all seven sites were located in the surface of the head of HA. Moreover, except for positions 218 and 133, five other sites were very close to the receptor binding site (RBS). Notably, position 234 was exactly sited in the RBS. Such locations strongly indicated that the seven mutations identified here may affect the antigenicity or pathogenesis of H9N2. Interestingly, MAb 2G10 also showed low HI titers against m3F2 mutant (Table 1), suggesting the N166D mutation may significantly alter the antigenicity of H9N2.

### 3.3. Single N166D Mutation Did not Affect the Viral Replication in MDCK Cells

**Figure 2:**
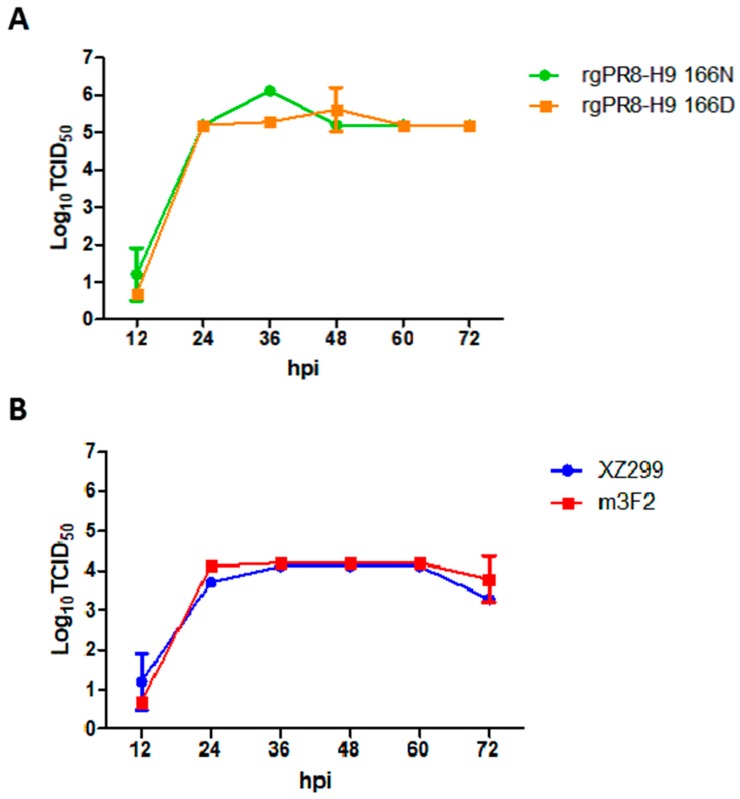
Viral growth kinetics in MDCK cell. MDCK cells were infected with rgPR8-H9 166N (**A**), rgPR8-H9 166D (A), XZ299 (**B**), and m3F2 (B) at an MOI of 0.001, and the tissue culture supernatants were collected at 12, 24, 36, 48, 60, and 72 hpi for the virus titers by TCID_50_ assays.

To investigate the impact of the N166D mutation on the antigenicity and pathogenesis of H9N2, two viruses, rgPR8-H9 166N and rgPR8-H9 166D, were rescued and the viral growth kinetics were performed. As shown in Figure 2A, the peak of viral titer for rgPR8-H9 166N was slightly higher than that for rgPR8-H9 166D. At other time points, the two viruses were similar to each other in virus titers. In addition, the viral growth kinetics for wild type virus XZ299 and mutant m3F2 showed that the ability of viral replication of XZ299 was very similar to that of m3F2 in MDCK cells (Figure 2B). These results demonstrate that the N166D mutation in H9 does not affect the viral replication of H9N2 in MDCK cells.

### 3.4. Single N166D Mutation Attenuated the Pathogenesis H9N2 in Mice

**Figure 3:**
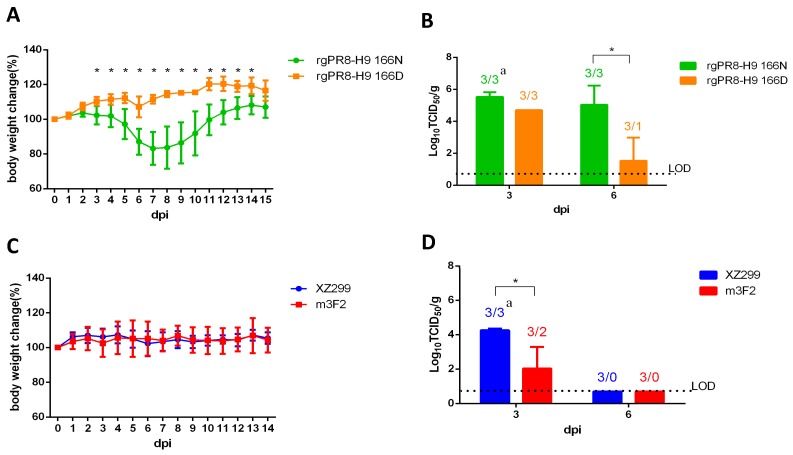
Infection study in mouse. Six-week-old Balb/c mice were inoculated with 10^5^ TCID_50_ of rgPR8-H9 166N, rgPR8-H9 166D, XZ299 and m3F2 respectively. (**A**,**C**) Percentage change of bodyweight in the infected mice daily; (**B**,**D**) virus load in lungs of the infected mice at 3 and 5 dpi. ^a^ The number of the total sample/the number of the virus positive sample. * *p* < 0.05.

To evaluate effect of N166D on the viral pathogenesis of H9N2, mice were infected with rgPR8-H9 166D, rgPR8-H9 166N, XZ299, and m3F2 respectively. As shown in Figure 3A, no clinical signs of disease or no body weight loss were observed in the mice infected with rgPR8-H9 166D, XZ299 or m3F2. However, the mice infected with rgPR8-H9 166N had a body weight loss and became weak from 5 dpi, and the maximum loss in the mice infected with rgPR8-H9 166N was about a 20% reduction in average body weight at 7 dpi. The attenuation in the viral pathogenesis due to the N166D mutation was also reflected by less viral shedding in the lungs of the mice infected with rgPR8-H9 166D vs. rgPR8-H9 166N (Figure 3B). Of note, the viral loading in the lungs from mice infected with XZ299 was also significantly higher than that in mice infected with m3F2 at day three post-infection as shown in Figure 3D. These observations suggest that the N166D mutation in HA of H9N2 attenuates the viral pathogenesis in mice.

### 3.5. Single N166D Mutation Resulted in Low HI Reaction for Chicken Sera

**Figure 4:**
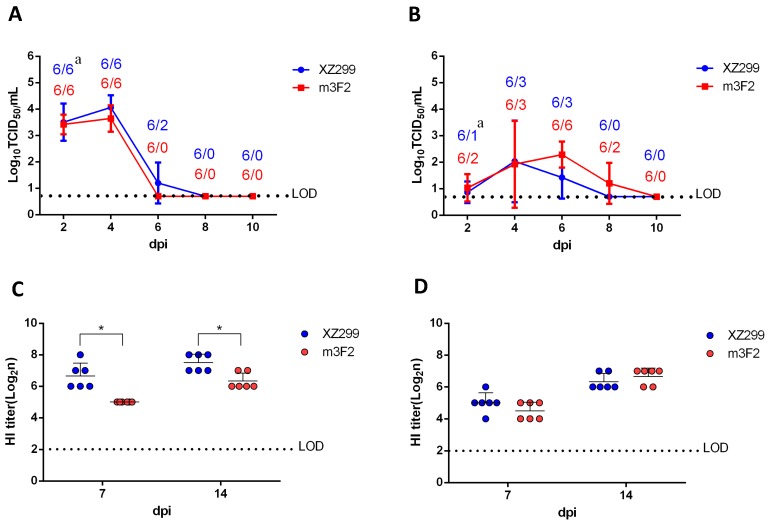
Infection study in chicken. Two-week-old chickens were inoculated with 10^5^ TCID_50_ of XZ299 and m3F2. At 2, 4, 6, 8, and 10 dpi, the swab samples of the larynx (**A**) and cloaca (**B**) were collected from six chickens in each group and titrated for the virus shedding by TCID_50_ in MDCK cells. At 7 and 14 dpi, six chickens from each group (**C**,**D**) were bled to analyze HI titers against XZ299 and m3F2. ^a^ The number of the total sample/the number of the virus positive sample. * *p* < 0.05.

To further investigate whether N166D affects antibody response and pathogenesis in chickens, chickens were infected with XZ299 and m3F2. As shown in Figure 4A,B, the laryngeal virus shedding in XZ299 group was slightly higher than that in m3F2 group whereas more chickens in m3F2 group shed viruses in the cloaca compared to XZ299 group. Notably, the sera of the infected chickens in XZ299 group had higher HI titers against XZ299 than those in m3F2 group but had HI titers against m3F2 similar to those in m3F2 group (Figure 4D,E). In addition, the HI titers of sera from XZ299 group against XZ299 were higher than those from m3F2 group against m3F2. These data demonstrate that although N166D mutation does not significantly affect the viral replication and shedding in the infected chickens but does result in the low HI antibody response.

## 4. Discussion

Mutations in the HA gene of influenza virus can affect both viral antigenicity and pathogenesis, and some HA mutants can efficiently escape from natural or vaccine-induced immunity, which leads to vaccination failure in the field [3,8,11]. This is the case with H9N2 virus in poultry industry of China. In this study, three MAbs against HA of a H9N2 strain A/chicken/Jiangsu/XZ299/2016 (XZ299) were generated and used for screening immune escape mutants. A total of four escape mutants with mutations across seven different amino acid residues were identified. The Q133L and N218D mutations are novel, whereas N166D, N167K, A168L, D207N, and L234M were previously identified [13,14]. All the Q133L/D207N/N218D mutations are present in the m2G10-1, m2G10-2 and m5C7 mutants, suggesting that MAb 2G10 and 5C7 could recognize similar antigenic epitopes. However, MAb 2G10, but not 5C7, had a weak HI reaction with the m3F2 mutant, which indicated that the epitopes recognized by MAb 2G10 and 5C7 were not the same, and the single N166D mutation in m3F2 might greatly affect antigenicity of H9N2 virus.

Although N166D mutation has been identified by others in the mutants with multiple mutations [13,14], the effects of the single mutation N166D on the antigenicity and pathogenesis of H9N2 remain unclear. In this study, the wild type virus XZ299, the mutant m3F2 and two rescued viruses (rgPR8-H9 166N and rgPR8-H9 166D) were used for evaluating the effects of N166D mutation in vivo. Since we could not efficiently rescue the recombinant virus with HA and NA of the XZ299, and six internal genes of PR8, the recombinant viruses rgPR8-H9 166N and rgPR8-H9 166D carried the HA of XZ299 or m3F2, and seven internal genes of PR8. Although the NA or the gene constellation of the virus can affect the viral ability to replicate or pathogenesis, there is only one amino acid difference between rgPR8-H9 166N and rgPR8-H9 166D. Therefore, the different phenotypes between rgPR8-H9 166N and rgPR8-H9 166D should be resulting from the N166D mutation. Although the mice infected with XZ299, m3F2 or rgPR8-H9 166D did not show any clinical signs, the mice infected with rgPR8-H9 166N did exhibit a significantly body weight loss. That XZ299 and m3F2 viruses did not cause disease in the infected mice is possibly due to their inadaptation to mice. The higher virus load in the lungs of the mice infected with rgPR8-H9 166N vs. rgPR8-H9 166D was consistent with the higher pathogenesis of rgPR8-H9 166N vs. rgPR8-H9 166D in mice. In the experiments with chicken, both viruses XZ299 and m3F2 caused a similar virus shedding in the larynx and cloaca. However, the sera of the chickens separately infected with one of the two viruses were different in the HI titers against the viruses. Compared to the sera of the chickens infected with XZ299, the sera of the chickens infected with m3F2 had a weaker HI reaction with both XZ299 and m3F2. Moreover, the sera of the chickens infected with XZ299 exhibited lower HI titers against m3F2 than XZ299. The HI data indicated that N166D mutation in HA did not only enable H9N2 virus escape from monoclonal antibody, but also led to weak antibody response in vivo or weak HI reaction with polyclonal antibody. As large-scale sequence analysis revealed, the field strains of H9N2 virus with N166D were mainly isolated in or near the countries with H9N2 vaccination, such as mainland of China, Vietnam and Egypt. This assay suggested that H9N2 with N166D in the field might result from its ability to escape from vaccination. Interestingly, there was also about 7% fraction of N166S strains which do appear distributed more globally (including Europe and North America). We also checked the HA sequence of H9N2 from wild birds and found that 28.57% HA of wild bird H9N2 (10/35) from China carried 166D, whereas no HA of wild bird H9N2 (0/78) from other country or region carried 166D. Of note, 9 of 10 H9N2 with HA carrying 166D are from wild waterfowl in Dongting Lake in China which isolated in 2012. In these H9N2 isolates from wild birds, 11.5% (13/113) and 65.49% (74/113) of HA had 166N and 166S, respectively. This all suggests that the immune escape or host adaptation driven or selected the variation of the site of 166 in the HA. It remains to be established how N166S mutation affects both antigenicity and pathogenicity of H9N2 strains.

In summary, this study is the first demonstration that a single mutation N166D in the escape mutant m3F2 has effects on the antigenicity and pathogenesis of H9N2. Although the N166D mutation attenuates the RP8 backbone-based virus other than the H9N2 backbone-based virus in mice, the N166D mutation does cause a weak antibody response in the chicken. The frequent isolation of the field H9N2 mutants with N166D in the countries with H9N2 vaccination highlights the significance of surveillance on such mutation and development of an efficient H9N2 vaccine.

## Figures and Tables

**Table 1:** Amino acid mutations in the hemagglutinin (HA) of escape mutants derived from A/chicken/Jiangsu/XZ299/2016/H9N2 (XZ299).

MAb	HI Titers (log_2_) ^a^					Mutations
	XZ299	Mutants	m3F2	rgPR8-H9 166N	rgPR8-H9 166D	
2G10	10	3 (m2G10-1)	4	9	1	Q133L/D207N/N218D/L234M ^b^
		- (m2G10-2)				Q133L/A168D/D207N/N218D
3F2	7	1 (m3F2)	1	10	-	N166D
5C7	10	5 (m5C7)	10	/ ^c^	/	Q133L/N167K/D207N/N218D

^a^ Shown are the titers with each MAb; -, no detected inhibition in hemagglutination inhibition (HI) assay. ^b^ Amino acid mutations are numbered according to the H9 number. ^c^ Not test.
